# Cost Estimation of Polymeric Adsorbents

**DOI:** 10.3390/polym11050925

**Published:** 2019-05-27

**Authors:** Despina A. Gkika, Efstathios V. Liakos, Nick Vordos, Christina Kontogoulidou, Lykourgos Magafas, Dimitrios N. Bikiaris, Dimitrios V. Bandekas, Athanasios C. Mitropoulos, George Z. Kyzas

**Affiliations:** 1 Complex Systems Lab, Department of Physics, International Hellenic University, GR-654 04 Kavala, Greece; despinagkika@gmail.com (D.A.G.); lmagafas@otenet.gr (L.M.); 2 Department of Physics, International Hellenic University, GR-654 04 Kavala, Greece; vordosn@yahoo.com (N.V.); dbandek@teiemt.gr (D.V.B.); 3 Hephaestus Advanced Laboratory, Department of Chemistry, International Hellenic University, GR-654 04 Kavala, Greece; stathilas@gmail.com (E.V.L.); amitrop@teiemt.gr (A.C.M.); 4 Department of Business Administration, University of Piraeus, 18536 Piraeus, Greece; ckonto@unipi.gr; 5 Laboratory of Polymer Chemistry and Technology, Department of Chemistry, Aristotle University of Thessaloniki, GR-541 24 Thessaloniki, Greece; dbic@chem.auth.gr

**Keywords:** adsorbent materials, polymers, chitosan, cost, raw material cost, labor cost, energy cost

## Abstract

One of the most promising techniques of recent research is adsorption. This technique attracts great attention in environmental technology, especially in the decontamination of water and wastewaters. A “hidden” point of the above is the cost of adsorbents. As can be easily observed in the literature, there is not any mention about the synthesis cost of adsorbents. What are the basic criteria with which an industry can select an adsorbent? What is the synthesis (recipe) cost? What is the energy demand to synthesize an efficient material? All of these are questions which have not been answered, until now. The reason for this is that the estimation of adsorbents’ cost is relatively difficult, because too many cost factors are involved (labor cost, raw materials cost, energy cost, tax cost, etc.). In this work, the first estimation cost of adsorbents is presented, taking into consideration all of the major factors which influence the final value. To be more comparable, the adsorbents used are from a list of polymeric materials which are already synthesized and tested in our laboratory. All of them are polymeric materials with chitosan as a substrate, which is efficiently used for the removal of heavy metal ions.

## 1. Introduction

One of the most promising techniques of recent research is adsorption. This technique attracts great attention in environmental technology, especially in the decontamination of water and wastewaters. It is considered that adsorption is one of the most efficient techniques for limiting or even completely cleaning water and “heavy” industrial effluents. The materials used in these techniques (namely adsorbent materials) can be easily applied in the tertiary stage of the biological treatment or water-cycle, having an ultimate target of capturing and binding the last remaining pollutants existing in the liquid phase (heavy metal ions, dyes, organic macromolecules, pharmaceutical compounds, etc.) of this (last) stage. The potential of this technique is so strong that more-and-more adsorbents are tested in order to find the most suitable one for further examination. The common question now is why adsorption is so promising, but the answer is easy if we look at the classic “bibliographic” advantages of this. Adsorption is an easy method, which can be applied without any special instrumentation, only some large-scale industrial adsorption beds (e.g., fixed-beds/columns) are required. Moreover, this technique is simple and fast, while the materials used can be easily reused after appropriate and simple pre-treatment (usually with aqueous eluants or some common organic solvents (acetone, methanol, etc.)).

Nowadays, a wide range of these types of adsorbents are extensively used, initially in lab-scale experiments for optimization and then in real industrial processes. Activated carbon, inorganic oxides, agricultural wastes, clays, and carbons are some of adsorbents used. However, special attention has recently been given to the use of polymeric materials because they can easily be modified based on the need of the pollutant. For example, if the pollutant is an anionic dye, the polymer can be grafted with positively charged groups (amino groups) to improve the attraction and consequently the adsorption ability. Grafting co-polymerization is a polymerization technique that allows for adding functional groups to chitosan. It can occur by utilizing a variety of options, such as free radicals, radiation, and enzymes [1,2]. The materials used for the polymerization should be able to carry reactive groups that can cause radical polymerization, which practically consists of a structure combining carbon and carbon atoms, or carbon and heteroatoms. Various types of vinyl, not limited to just acrylic acid, acrylamide, and vinyl pyridine, have been mentioned in prior art for chitosan grafting. The effectiveness of grafting appears to vary based on the selected grafting method and chosen parameters.

A “hidden” point of all the above is the cost of adsorbents. As can be easily observed in the literature, there is not any mention about the synthesis cost of adsorbents. What are the basic criteria with which an industry can select an adsorbent? What is the synthesis (recipe) cost? What is the energy demand to synthesize an efficient material? All of these are questions which have not been answered, until now. The reason for this is that the estimation of adsorbents cost is relatively difficult, because too many factors are involved (labor costs, raw materials cost, energy cost, tax cost, etc.).

The novelty of the present work is clear, we present the first estimation cost of adsorbents, taking into consideration the cost factors which influence the final value. To be more comparable the adsorbents used are a list of polymeric materials which are already synthesized and tested in our laboratory. All of them are polymeric materials with chitosan as a substrate, which is efficiently used for the removal of heavy metal ions. Chitosan-based materials were selected because this polymer (poly-β-(1→4)-2-amino-2-deoxy-D-glucose) is a nitrogenous (amino-based) polysaccharide, which is produced in large quantities by *N*-deacetylation of (its origin compound) chitin. The major advantage of chitosan is the existence of modifiable positions in its chemical structure. This gives chitosan the opportunity to be employed in different applications [3,4,5,6,7,8,9,10,11,12]. The modification of the chitosan molecule with (i) grafting (insert functional groups) or (ii) cross-linking reactions (unite the macromolecular chains with each other) leads to the formation of chitosan derivatives with superior properties, such as the enhancement of adsorption capacity and resistance in extreme media conditions, respectively.

## 2. Materials and Methods

Large-scale data was gathered during the initial stage of the research process. The top-down approach was utilized to assess and verify the chitosan market share. Multiple studies and information published in various sources (scientific papers, articles, industry reports etc.) were taken into account while performing the secondary research. The process involved the use of multiple other avenues of information such as directories and databases to identify and obtain information for the purposes of more technical and market-oriented research on the chitosan market. This work explores the current market trends, analyzes past data from 2010 to 2018, and discusses the forecasts for the following years, ranging from 2020 to 2025.

### 2.1. Recipe Cost

The data was obtained from various sources, for the most part via in-person interviews with the corresponding managers of two Greek Chemistry Laboratories. They were selected due to their experience with the subject matter. The main point of the interviews was the establishment and assessment of a list of attributes of “popular” adsorbents.

Conducting interviews in person offers the advantage of observing the interviewee’s reactions, as well as listening to their replies unobtrusively. Our approach was based on unstructured interviews because of their flexibility for all people involved, as suggested by Gubrium and Holstein [13]. In addition, there is also the opportunity to explore select issues of importance [14,15]. These select topics take advantage of the interviewee’s expertise on certain topics [16]. The main concern in these situations is to properly balance appropriate reporting of research results and the occasional risk of revealing interviewees’ personal information, as opposed to higher confidentiality with the risk of results getting disputed [17]. The confidentiality is important to protect the participants as remaining anonymous allows these experts to express their opinions freely [18]. For the current work, the participants are male professors. The interview was comprehensive, lasting more than 40 minutes. Based on the replies, we deducted what the most important attributes affecting costs are, namely the raw materials and the adsorption energy costs.

#### Recipe Selection

In an effort to explore the adsorption phase in more depth and to uncover the factors that characterize the adsorbents, we conducted a literature search to identify and further analyze their recipes. The most cited ones were selected, so that the corresponding cost factors could be evaluated. 

### 2.2. Raw Material Cost

Raw material cost prices for the study of chitosan were obtained from publicly released catalogues. It should be noted that chitin and chitosan products can have very wide price ranges, depending on the quality of the final product. For example, chitosan prices might range between United States Dollar (USD) 10 to USD 1000 per kilogram. The costs calculated in this work are all translated to euros (€). The analysis took into account multiple market prices, as provided by vendors all over the world. The raw material cost for each case included the adsorbent cost as well as the metal recovery cost.

### 2.3. Energy Cost

The energy cost corresponds to the energy spent for the various stages of the adsorption process. Electricity costs per KWh used are based on the average energy price in Greece for 2019 (0.194 Euro/KWh). This price was retrieved by the Hellenic Public Power Corporation S.A. [19]. Therefore, the energy cost in Euros has been estimated as the product of the amount of KWh spent and the price of 1 KWh in Greece.

### 2.4. Labor Cost

This type of cost consists of the compensation of researchers participating in the project, with the addition of taxes and benefits. For the purposes of this study, the personnel required for the synthesis process comprises of 1 researcher working for 1 work-day (i.e., 3 h). The average wages of the personnel were assessed based on information from Glassdoor [20], which maintains a rich database with employee wages per company and country, depending on the position.

## 3. Results and Discussion

Chitosan has multiple applications across various industrial sectors which renders it quite attractive for investors. The high cost of production, however, is a significant limiting factor. Researchers are focusing on making it more cost effective, which can also be achieved if we can reach economy of scale. On this basis, two major modifications can be found in the literature: (i) grafting reactions to enhance the adsorption capacity, and (ii) cross-linking reactions to make the final product more rigid to extreme conditions (pH and salinity) and increase the reusability.

Grafting cationic groups into chitosan has been utilized for a variety of applications. It has been successful in removing anionic pollutants. Li et al. [21] produced chitosan adsorbents altered by a quaternary ammonium salt that is used for removing methyl orange and Cr(VI). The outcomes suggest that the strong cationic groups that enriched chitosan displayed improved adsorption ability for both Cr(VI) as well as methyl orange compared to pure chitosan. Furthermore, the results also indicated that the adsorption capacity was significantly influenced by the pH levels, suggesting that the electrostatic attractions were an important part of the process. The use of an amino group to enrich chitosan is thus deemed as accountable for the adsorption of metal ions.

Raw chitosan [22] is limited significantly due to its high solubility in most mineral and organic acid solutions, which makes the assessment of its use as a sorbent for the treatment of industrial effluents particularly difficult. A successful way to overcome this and enhance its chemical stability in acidic conditions is through cross-linking. Various popular cross-linking agents, such as glutaraldehyde (GLA) and epichloridrin (EPI), have been employed to achieve this. 

The main contributors of growth for the chitosan market are the high availability of raw materials and the emerging applications and applicability in multiple sectors. Thorough research and development activity on pre-existing technology is yet another reason for a market-size growth, despite the expensive production process of high-quality chitosan. It should be noted that occasional product inconsistency due to seasonal, regional, or chemical modifications might hinder the market growth to a certain degree [23].

It should be noted that the adsorption ability of the materials mentioned in this work also rely on other experiment conditions including pH levels, starting concentration level, contact duration, dosage, and competitive ion presence. Wan Ngah and Fatinathan were successful in cross-linking chitosan beads with GLA as a means of removing Cu(II) ions in aqueous solutions [24]. The cross-linked chitosan was not soluble in acetic acid solution, which verified that cross-linking improved the resistance of the polymer to the acid. The effect of GLA in adsorption was also studied. The use of cross-linkers or even the increased amount of those agents had a negative effect on the adsorption ability due to the reduced number of amino groups serving as binding sites for the metal ions.

**Figure 1:**
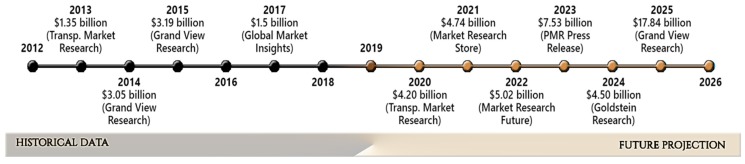
Historic evolution of chitosan market prices and projections for the future, based on published market reports.

According to Transparency Market Research [25], globally, the chitosan market size in 2013 was estimated to be 1.35 million, reaching 4.20 billion by 2020 (Figure 1). Grand View Research [26] estimated 3.19 billion for 2015 and projected it would reach 17.84 billion by 2025.

Global Market insights published and estimate of 1.5 billion for 2017 [27]. Market Research Store projected chitosan would reach 4.74 billion by 2021, while for the following years (2022 and 2023), Market Research Future [28] and PMR Press Release [29] projected 5.02 and 7.53 billion, respectively.

For comparison purposes, the outcomes of this work, along with information about the adsorption capacities of a range of chitosan-based adsorbents from the literature, are presented in Table 1.

Table 1 shows the limited comparability of the final products. If anyone changes one of the synthesis parameters (time, amounts, reagents, etc.), the final product will be different, so therefore, the adsorption evaluation will vary. Hence, it is not easy to compare adsorbents of different sources. Kyzas et al. [30] showed that the GLA-chitosan presented higher capacities for Cu(II) removal when the grafting agent used was poly(acrylic acid) and not poly(acrylamide). The latter depends on the “nature” of attractions among active adsorption sites of chitosan and the charged copper ions. For this reason, the value of pH was added to Table 1 in order to clarify the surficial charge of the material. In the same study [30], the removal of Cr(VI) was higher, by using GLA-chitosan grafted with poly(acrylamide) rather than poly(acrylic acid), confirming the above-mentioned finding. Table 1 also presents many examples of differentiations due to the adsorbent–adsorbate interactions.

### Calculations

To calculate the recipe cost, Table 2 was drawn, gathering all appropriate information. In this Table, the method and duration used during synthesis were presented along with the relative instrumentation (reported energy consumption). Furthermore, Table 3 summarizes the costs estimated for raw materials, energy, and presents the sum of them. The latter will be from hereafter the basis on which we can compare the cost of each polymeric adsorbent in this study. The equation used for calculations is:

(1) $$E_{c}=P_{D}\cdot a\cdot t\cdot C_{c}$$

where E_c_ is the energy cost (€), P_D_ is the power consumed by the device (kW), a is a load factor (if we use the device in full mode then a = 1, while for half mode a = 0.5, t is the usage of the device (h), and C_c_ is the energy cost (€/KWh).

According to Table 3, the most expensive adsorbent produced is the grafted derivative of chitosan with succinic anhydride (21.89 €), in which the basic source is the energy demand with increasing time of stirring and freeze-drying (14.24 €), which is double the raw materials cost (7.65 €). On the other hand, the second most expensive polymer studied was the grafted chitosan derivative of poly(ethylene imine) (17.13 €), which is majorly attributed to the cost of raw materials and especially PEI (55.90 €/100 mL), almost half the price is originated from the raw materials cost (8.09 €). It is worth mentioning that the composite of graphite and magnetic chitosan presented the lowest energy demand (3.76 €) based on the synthesis procedure, which led to the lowest final estimated cost (6.16 €).

Recipe costs depend on a variety of factors, ranging from current research needs, expertise, and appropriate recipe selection. For example, the same material could be evaluated differently, because an experienced researcher will know how to find and select a lower cost recipe, without undermining the final product quality.

**Figure 2:**
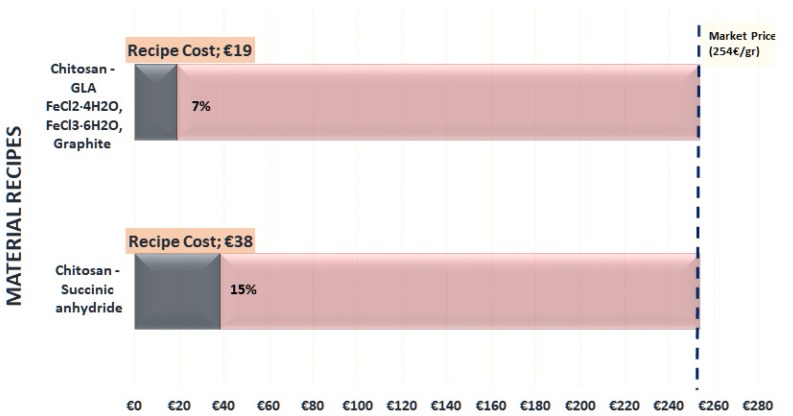
Recipe cost versus Market price.

Figure 2 depicts the comparison of the cost distribution of the cheapest and most expensive chitosan synthesis recipes explored in this work, as it compares to the market price of high purity-derived Chitosan. The market price could reach 254 €/g and it far outweighs the recipe costs calculated, consisting of the raw material, energy, and labor costs.

The cost analysis for any technology is the most common form of cost evaluation as it basically takes into account all of the costs (direct or not) of a product throughout its lifecycle. The labor and energy costs take up a very significant percentage of the final cost of the chitosan synthesis process. Almost 70% of the total recipe cost is labor cost. Raw materials comprise 23% of the total recipe cost. It is noted that the energy cost does not surpass 45% of the final cost. It therefore becomes obvious that the labor cost is the most significant cost factor of the recipe process for chitosan. This recipe cost is far higher than the material’s market price, which currently spans between. 

**Figure 3:**
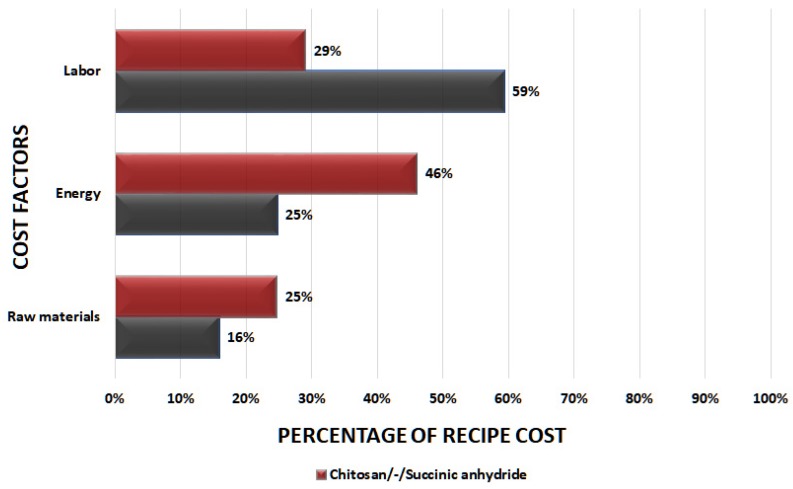
Distribution of recipe cost factors for different chitosan recipes.

Operational costs were the main factors affecting the recipe cost evaluation. These include raw materials, personnel wages, and energy consumed. Figure 3 depicts the cost factors for the studied Chitosan recipes as percentages of the total recipe cost. Prior research indicated that usually the raw material cost is the highest cost driver, however, in the case of these chitosan recipes, due to the comparatively cheaper materials, it is actually the labor and energy costs that appear as the most significant. The energy cost is particularly high due to the energy requirements for the pyrolysis stage. 

Raw materials comprise about 16% and 25% of the total recipe cost, respectively. It is of note that for each recipe the major cost driver is different. In the case of Chitosan/GLA/FeCl_2_·4H_2_O, FeCl_3_·6H_2_O, Graphite, the most significant cost is Labor, reaching almost 60%, while for the case of Chitosan/-/Succinic anhydride the most important cost driver is Energy, reaching about 46%. The labor cost is the highest percentage overall in both recipes, but it is evident that depending on the material produced, the balance of costs might change, favoring different factors. For both cases of this study, the recipe cost was actually lower than the market price of high-purity non-animal-derived Chitosan, which is about 254 € per gram.

The ever-increasing demand for green materials has led to the growth of the polymer industry. Polymers have a wide variety of applications, but their high cost has been a hindrance, hence a lot of research has been devoted to cost reduction and selection of "the greenest". Chitosan is one of the polymers found in nature in abundance. Its cost is relatively low but depends on the specific physicochemical properties required for the target application. In this case, the raw material cost does not really affect the final total recipe cost.

In order to limit the current and future recipe costs without sacrificing the quality, new strategies must be forged. Our results suggest that the main cost factors are labor and energy. Identifying fixed and variable costs will assist in optimizing them. Labor cost is a variable cost. In order to minimize it as much as possible, researchers could be trained to perform both the synthesis and characterization efficiently. Furthermore, depending on materials used, an additional step would be to select equipment that would minimize energy consumption by performing multiple processes concurrently.

## 4. Conclusions

Chitosan has a great potential as a sorbent with the ability to remove a variety of contaminants, however its limitations require adjustments to be made to enhance its abilities. Raw chitosan has a crystalline structure, which lowers its adsorption ability due to the adsorption taking place on the crystal part. Cross-linking groups tend to react with the amine parts of chitosan, which are known as common bonding sites for metal ions. It is thus important to proceed in the grafting of functional groups on chitosan, in an effort to enhance selectivity and efficiency. Grafting seems like a suitable approach for refining the intrinsic attributes of natural polymers or for enriching them with new ones. Cross-linking and grafting are great alternative options for establishing an adsorption system. The effect of cross-linked content on the process or the actual properties of chitosan should be further explored in future research on discovering more efficient adsorbents.

## Figures and Tables

**Table 1:** Synthesis recipes of metals adsorption using various polymeric chitosan-modified materials (dosage: 1 g of adsorbent per 1 L of adsorbate solution).

Polymer	Modification Agents		pH	Adsorption Capacity (mg/g)	Metal Ion	Ref
	Cross-Linker	Grafting Agent				
Chitosan	GLA	-	6	208	Cu(II)	[30]
Chitosan	GLA	poly(acrylic acid)	6	318	Cu(II)	[30]
Chitosan	GLA	poly(acrylamide)	6	166	Cu(II)	[30]
Chitosan	GLA	-	4	655	Cr(VI)	[30]
Chitosan	GLA	poly(acrylic acid)	4	518	Cr(VI)	[30]
Chitosan	GLA	poly(acrylamide)	4	935	Cr(VI)	[30]
Chitosan	GLA	-	5	145	Hg(II)	[31]
Chitosan	GLA	FeCl_2_·4H_2_O, FeCl_3_·6H_2_O	5	152	Hg(II)	[31]
Chitosan	GLA	Poly(ethylene imine)	4	125	Cr(VI)	[32]
Chitosan	GLA	Graphite oxide	6	381	Hg(II)	[33]
Chitosan	GLA	FeCl_2_·4H_2_O, FeCl_3_·6H_2_O, Graphite oxide	6	397	Hg(II)	[33]
Chitosan	-	-	5	167	Zn(II)	[34]
Chitosan	-	Succinic anhydride	5	245	Zn(II)	[34]
Chitosan	EPI	Chlorosulfuric acid	6	85	Ni(II)	[35]
Chitosan	EPI	Chlorosulfuric acid	6	76	Hg(II)	[35]
Chitosan	GLA	Poly(ethylene imine)	6	152	Ni(II)	[35]
Chitosan	GLA	Poly(ethylene imine)	6	126	Hg(II)	[35]

**Table 2:** Synthesis recipes of metals adsorption using various polymeric chitosan-modified materials (dosage: 1 g of adsorbent per 1 L of adsorbate solution).

Polymer	Cross-Linker	Grafting Agent	Method	Duration (h)	Instrumentation	Ref
Chitosan	GLA	-	Soxhlet	24	Soxhlet Electrothermal (580 W)	[30]
			Vacuum Drying	12	Oven Thermofisher (1,45 kW)	
			Stirring	1	Stirrer CAT M 6,1 (580 W)	
Chitosan	GLA	poly(acrylic acid)	Soxhlet	24	Soxhlet Electrothermal (580 W)	[30]
			Vacuum Drying	12	Oven Thermofisher (1,45 kW)	
			Stirring	3	Stirrer CAT M 6,1 (580 W)	
Chitosan	GLA	poly(acrylamide)	Soxhlet	24	Soxhlet Electrothermal (580 W)	[30]
			Vacuum Drying	12	Oven Thermofisher (1,3 kW)	
			Stirring	1	Stirrer CAT M 6,1 (580 W)	
Chitosan	GLA	-	Soxhlet	24	Soxhlet Electrothermal (580 W)	[31]
			Drying	24	Oven Thermofisher (1,45 kW)	
			Stirring	3	Stirrer CAT M 6,1 (580 W)	
Chitosan	GLA	FeCl_2_·4H_2_O, FeCl_3_·6H_2_O	Stirring	4	CAT M 6,1 (580 W)	[31]
			Freeze-drying	12	Christ Alpha 1-4 (510 W)	
			Sonication	0.5	Sonicator Fisherbrand (500 W)	
			Vacuum Oven	12	Oven Thermofisher (1,45 kW)	
Chitosan	GLA	Poly(ethylene imine)	Soxhlet	24	Soxhlet Electrothermal (580 W)	[32]
			Vacuum Drying	12	Oven Thermofisher (1,45 KW)	
			Stirring	29	CAT M 6,1 (580 W)	
Chitosan	GLA	Graphite oxide	Soxhlet	24	Soxhlet Electrothermal (580 W)	[33]
			Vacuum Oven	36	Oven Thermofisher (1,45 kW)	
			Ultrasonic Stirring	0.5	Sonicator Fisherbrand (500 W)	
			Stirring	7.5	CAT M 6,1 (580 W)	
Chitosan	GLA	FeCl_2_·4H_2_O, FeCl_3_·6H_2_O	Stirring	3	CAT M 6,1 (580 W)	[33]
		Graphite oxide	Sonication	0.5	Sonicator Fisherbrand (500 W)	
			Vacuum Oven	12	Oven Thermofisher (1,45 kW)	
Chitosan	-	-	Stirring	1	CAT M 6,1 (580 W)	[34]
			Oven	12	Oven Thermofisher (1,45 KW)	
			Soxhlet Washing	24	Soxhlet Electrothermal (580 W)	
Chitosan	-	Succinic anhydride	Stirring	21	CAT M 6,1 (580 W)	[34]
			Freeze Drying	120	Christ Alpha 1-4 (510 W)	
Chitosan	EPI	Chlorosulfuric acid	Stirring	2	CAT M 6,1 (580 W)	[35]
			Oven Drying	24	Oven Thermofisher (1,45 kW)	
			Soxhlet Washing	24	Soxhlet Electrothermal (580 W)	
Chitosan	GLA	Poly(ethylene imine)	Soxhlet	24	Soxhlet Electrothermal (580 W)	[35]
			Stirring	17	CAT M 6,1 (580 W)	
			Oven Drying	24	Oven Thermofisher (1,45 kW)	

**Table 3:** Raw materials and energy costs in selected recipes.

Material Produced Polymer/Cross-Linker/Grafting Agent)	Raw Materials	Cost			Ref
		Raw Materials ^a^ (€)	Energy ^b^ (€)	Final Recipe (€)	
Chitosan/GLA/-	Chitosan (High molecular weight); Glutaraldehyde (50 wt% in water); Acetic acid solution (>99%)	2.32	6.19	8.51	[30]
Chitosan/GLA/Poly(acrylic acid)	Chitosan (High molecular weight); Glutaraldehyde (50 wt% in water); Acetic acid solution; Potassium persulfate; Acrylic acid	2.92	6.41	9.33	[30]
Chitosan/GLA/Poly(acrylamide)	Chitosan (High molecular weight); Glutaraldehyde (50 wt% in water); Acetic acid solution; Potassium persulfate; Acrylamide	2.59	5.84	8.43	[30]
Chitosan/GLA/FeCl_2_·4H_2_O	FeCl_2_·4H_2_O (p.a > 99.0%); Chitosan (High molecular weight); FeCl_3_·6H_2_O (reagent grade, 97%); Glutaraldehyde (50 wt% in water; Acetic acid solution (>99%)	2.13	5.06	7.19	[31]
Chitosan/GLA/Poly(ethylene imine)	Chitosan (High molecular weight); Epichlorohydrine; Acetic acid solution (>99%); Poly(ethylene imine) (30%)	8.09	9.34	17.43	[32]
Chitosan/GLA/Graphite oxide	Chitosan (High molecular weight); Glutaraldehyde (50 wt% in water; Acetic acid solution (>99%); KMnO_4_ (>99.0%); Graphite flakes; H_2_SO_4_ (95%–98%); H_2_O_2_ (30wt%)	1.88	13.72	15.60	[33]
Chitosan/GLA/FeCl_2_·4H_2_O, FeCl_3_·6H_2_O, Graphite	FeCl_2_·4H_2_O (p.a > 99.0%); Chitosan (High molecular weight); FeCl_3_·6H_2_O (reagent grade, 97%); Glutaraldehyde (50 wt% in water; Acetic acid solution (>99%); KMnO_4_ (>99.0%); Graphite flakes; H_2_SO_4_ (95%–98%); H_2_O_2_ (30 wt%)	2.40	3.76	6.16	[33]
Chitosan/-/Succinic anhydride	Chitosan (High molecular weight); Acetic acid solution (>99%); Succinic anhydride; Methanol; Acetone	7.65	14.24	21.89	[34]
Chitosan/EPI/Chlorosulfuric acid	Dichloroacetic acid (>99%); Formamide (>99.5%); Chitosan (High molecular weight); Epichlorohydrine; Acetic acid solution (>99%)	3.22	9.68	12.90	[35]

^a^ per 1 g of final product; ^b^ 1 KWh = 0.194 €.
